# Automated Analysis of Fluorescence Microscopy Images to Identify Protein-Protein Interactions

**DOI:** 10.1155/IJBI/2006/69851

**Published:** 2006-08-22

**Authors:** S. Venkatraman, M. J. Doktycz, H. Qi, J. L. Morrell-Falvey

**Affiliations:** ^1^ Life Sciences Division, Oak Ridge National Laboratory, Oak Ridge, TN 37831, USA; ^2^ Department of Electrical and Computer Engineering, University of Tennessee, Knoxville, TN 37996, USA

## Abstract

The identification of protein interactions is important for elucidating biological networks. One obstacle in comprehensive interaction studies is the analyses of large datasets, particularly those containing images. Development of an automated system to analyze an image-based protein interaction dataset is needed. Such an analysis system is described here, to automatically extract features from fluorescence microscopy images obtained from a bacterial protein interaction assay. These features are used to relay quantitative values that aid in the automated scoring of positive interactions. Experimental observations indicate that identifying at least 50% positive cells in an image is sufficient to detect a protein interaction. Based on this criterion, the automated system presents 100% accuracy in detecting positive interactions for a dataset of 16 images. Algorithms were implemented using MATLAB and the software developed is available on request from the authors.


Hindawi Publishing Corporation
International Journal of Biomedical Imaging
Volume 2006, Article ID 69851, Pages 1–10
DOI 10.1155/IJBI/2006/69851

### Automated Analysis of Fluorescence Microscopy Images to Identify Protein-Protein Interactions

**S. Venkatraman,[1,2] M. J. Doktycz,[1] H. Qi,[2] and J. L. Morrell-Falvey[1]**

[1] *Life Sciences Division, Oak Ridge National Laboratory, Oak Ridge, TN 37831, USA*
[2] *Department of Electrical and Computer Engineering, University of Tennessee, Knoxville, TN 37996, USA*

Received 6 March 2006; Revised 13 June 2006; Accepted 26 June 2006

Recommended for Publication by Vasilis Ntziachristos

The identification of protein interactions is important for elucidating biological networks. One obstacle in comprehensive interaction studies is the analyses of large datasets, particularly those containing images. Development of an automated system to analyze an image-based protein interaction dataset is needed. Such an analysis system is described here, to automatically extract features from fluorescence microscopy images obtained from a bacterial protein interaction assay. These features are used to relay quantitative values that aid in the automated scoring of positive interactions. Experimental observations indicate that identifying at least 50% positive cells in an image is sufficient to detect a protein interaction. Based on this criterion, the automated system presents 100% accuracy in detecting positive interactions for a dataset of 16 images. Algorithms were implemented using MATLAB and the software developed is available on request from the authors.

Copyright © 2006 S. Venkatraman et al. This is an open access article distributed under the Creative Commons Attribution License, which permits unrestricted use, distribution, and reproduction in any medium, provided the original work is properly cited.

### 1. INTRODUCTION

Functional and location proteomics with their high content information are revolutionizing current research in the postgenomic era [1]. However, high-throughput imaging studies produce large volumes of information, rich data that can be both time consuming and cumbersome to analyze manually. Such studies would benefit from an effective processing/analytic system that can automatically exploit the copious information available in the acquired data.

The datasets generated by image acquisition systems can be analyzed using various image processing techniques to uncover vital information. Until recently, significant efforts have been channeled towards automating image analysis for applications involving machine vision and medical imaging modalities such as MRI, PET, and SPECT [2, 3]. However, fewer application examples exist in the field of optical microscopy of biological samples. Though image acquisition in this field has been automated for quite some time, the analysis domain typically relies on independent scorers to perform the task [4]. This is due to a variety of factors such as the inability of generic image processing algorithms to discover the rich information embedded in microscopy images and, more importantly, the risk of improper interpretation [5].

With the advent of high-throughput image collection and analysis tools, the field of systems biology has the potential to explore new dimensions. Fluorescence microscopy, along with the advances made in the software industry, has led to novel approaches for elucidating a wide range of features in the field of proteomics [1, 6]. Considering the large number of proteins, the study of protein localization is an application where automated image analytic solutions could enhance the speed and efficiency of the procedure.

In this paper, we evaluate and apply advanced image processing techniques targeted at identifying protein interactions by detecting patterns of protein localization within a bacterial cell. For this interaction assay, as illustrated in Figure 1, proteins of interest are fused to either green fluorescent protein (GFP), or DivIVA, a bacterial cell division protein from *Bacillus subtilis* that localizes to the cell poles [7] and co-expressed in *E. coli* cells [8, 9]. Upon induction of DivIVA-fusion protein expression, the GFP-fusion protein localizes to the cell poles if a positive interaction occurs. In the case of a negative result,the GFP-fusion protein remains diffusely localized in the cell. The objective of this work is to automate image analysis of protein localization patterns from a set of differential interference contrast (DIC) and fluorescence images. The decision rule for a positive interaction is known from results described in [8, 9].

Simultaneous assessment of multiple cells in a single field of view is needed to derive the statistical information required to confidently assign a positive or negative interaction score to each experiment. According to experimental studies fields containing at least 50% of cells displaying a localization pattern consistent with a positive interaction between the two proteins of interest are sufficient for assigning a positive score [10]. However, this procedure creates significant challenges for automated analysis. For example, closely spaced cells can be difficult to differentiate and lead to erroneous cell counts. Moreover, cells on the verge of dividing have unusual shapes which can confuse the assessment of GFP-fusion protein localization patterns. Another common problem with fluorescence microscopy images is the presence of unwanted fluorescence. This is sometimes referred to as “bleeding” and can lead to ambiguous results. Finally, the presence of inclusion bodies[1] needs to be distinguished from true sites of GFP-fusion protein localization. This paper discusses various techniques employed to overcome such problems in order to achieve unambiguous results from automated image analyses. Murphy and colleagues have described a set of subcellular location features for microscopy images aimed at automated classification of protein localization patterns in eukaryotic cells [11]. A few pertinent features from these studies along with a set of DIC images are used to identify positive interactions according to the decision rules reported in [8, 9]. We use a set of 16 DIC and corresponding fluorescence images to evaluate the proposed automated image analysis algorithm. Results from the automated algorithm are compared with the decisions made by an expert scorer. This evaluation further validates the effectiveness of the proposed system and its potential in analyzing a wide range of complex protein localization studies.

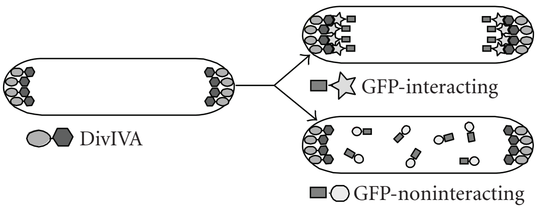

Figure 1: Cartoon of the assay under study; DivIVA directs localization of protein of interest 1 (POI1) to the poles. GFP-POI2 relocates to the cell poles if POI1 and POI2 directly interact. GFP-POI2 remains diffusely localized if no interaction occurs.

### 2. SYSTEMS AND METHODS

### 2.1. Sample preparation and image acquisition

*E. coli* strain BL21-DE3 (Invitrogen, Carlsbad, CA) was cotransformed with two vectors based on pBAD24 [12] and pACYC184 (New England Biolabs, Beverly, MA) encoding pairs of potentially interacting proteins from *Rhodopseudomonas palustris* fused to either DivIVA or GFP. Construction of these vectors will be described in detail elsewhere [10]. Briefly, the Gateway cassette from pDEST17 (Invitrogen) including the T7 promoter was PCR amplified and cloned into the *Hind*III site of pACYC184. GFP was then amplified and cloned into the unique *Nde*I site of the pACYC184-DEST17 modified plasmid to produce an *N*-terminal GFP-fusion protein after an LR recombination reaction (Invitrogen). Similarly, the Gateway cassette from pDEST14 and *DivIVA* from *Bacillus subtilis* genomic DNA were PCR amplified and cloned in frame into pBAD24 to produce an *N*-terminal DivIVA-fusion protein following an LR recombination reaction. The *R. palustris* gene products tested in this study are GroES2 (RPA2165) and GroEL2 (RPA2164) [13]. Cotransformed cells were grown for at least 6 hours at 30°C or 37°C in LB medium containing 50 $\mu$g/ml ampicillin and 15 $\mu$g/ml chloramphenicol to maintain plasmid selection and then imaged using a Leica SP2 confocal laser scanning microscope to determine the localization pattern of the GFP-fusion protein. After assessment of the baseline pattern of GFP localization, arabinose was added to the medium to a final concentration of 0.2% to induce expression of the DivIVA-fusion protein. The cells were incubated for an additional hour at 30°C or 37°C. Following induction of the DivIVA-fusion protein, the cells were imaged again to determine if a change in the pattern of GFP-fusion protein localization occurred. If the GFP-fusion protein was recruited to the cell poles following expression of the DivIVA-fusion protein, the data was interpreted as showing a positive interaction between the two proteins of interest. Images were collected using Leica confocal software (LCS). The basic methodology of sample handling and image acquisition are outlined in Figure 2.

To stain cell membranes, *E. coli* cells were grown in liquid LB medium as described above. Approximately 15 minutes prior to harvesting the cells, 200 ng/ml FM5-95 (Molecular Probes, Eugene, OR) was added directly to the culture to stain the membranes. The cells were then harvested by centrifugation, washed two times with 0.01 M phosphate buffered saline (pH 7.4), and prepared for microscopy.

### 2.2. Image processing algorithm development

Figure 3 shows a block diagram that describes the flow of different image processing steps implemented in our analyses. Owing to the visual similarity between images of inclusion bodies and those of a positive interaction, the same set of image processing and feature extraction steps are used to identify inclusion bodies before induction of DivIVA-fusion protein expression. Inclusion body identification will be further discussed in Section 3.

[1] Intracellular protein aggregates that are usually observed in bacteria upon protein over expression.

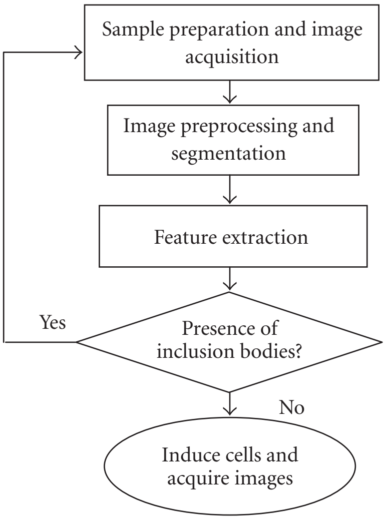

FIGURE 2: Basic methodology for sample handling and image acquisition.

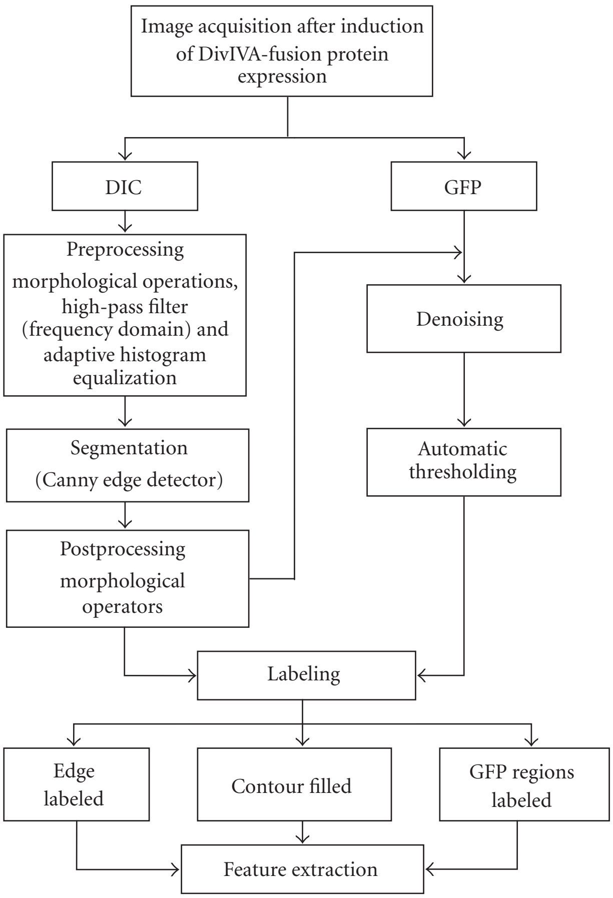

FIGURE 3: Image processing flow chart.

The image preprocessing and segmentation block in Figure 2 can be further divided into three general procedures: image enhancement (preprocessing), image segmentation, and postprocessing, and connected component labeling, as illustrated in Figure 3.

Upon acquiring the DIC and fluorescence images (GFP), the images are processed through two slightly different procedures because of their different intrinsic features. Preprocessing of DIC images consists of enhancing edge-based information along cell boundaries. Since we are interested in cell boundary information, we adopt the effective morphological operation, that is, finding the bottom-hat[2] version of the original image and subtract the same from the original. This would give us a steeper contrast along cell boundaries. To further improve this, we run it through a second-order Butterworth high-pass filter in the frequency domain. Finally, to enhance this contrast along cell boundaries and to make it sensitive enough for the following segmentation procedure, an adaptive histogram equalization function is used. We adopt the function provided by MATLAB which divides the image into tiles (size determined by the user); a monotonic, non-linear mapping is applied that reassigns intensity values of pixels in the input tile to create an output tile that contains a uniform distribution of intensities. This step results in a flat histogram. The tiles are then combined using bi-linear interpolation to form an output image. The advantage of using adaptive histogram equalization over tradition histogram equalization is that it avoids highlighting noise details in the image, thus improving the intensity difference along the boundaries. In order to avoid oversegmentation, an average filter with a $3 \times 3$ mask is used to connect segments of close distance. After enhancing the DIC images, a *Canny* edge detector (Canny, 1986) is used to identify the edges of individual cells. The standard deviation of the Gaussian filter ($\sigma$) determines the thickness of edges detected and is the only free parameter in the process of identifying cell contours. An evaluation is conducted in Section 3 to study the effect of $\sigma$ on the overall performance of the system. The cell contours are then filled using a binary fill option in MATLAB. We now apply morphological operators of opening and closing to remove any speckle noise from the binary image.

The fluorescence image, different from the DIC image, often contains background noise (Figure 4(a)), which can be mostly removed by keeping just the area occupied by the cell (Figure 4(b)). This can be obtained from the corresponding DIC image as explained above. An automated global threshold algorithm described by Otsu [14] is applied on the denoised fluorescence image to obtain a binary image. To include weak signals during thresholding, a value equal to one-thirds the value obtained by Otsu's method is used. This threshold is another free parameter that affects the algorithm sensitivity in determining the presence of positive localization patterns within the identified cell. This effect is evaluated in Section 3. This binary image is then passed through a combination of opening, closing, and a median filter to group neighboring pixels and to remove any speckle noise

[2] *Imbothat* is a morphological filter provided by MATLAB and uses morphological closing operation to output image troughs.

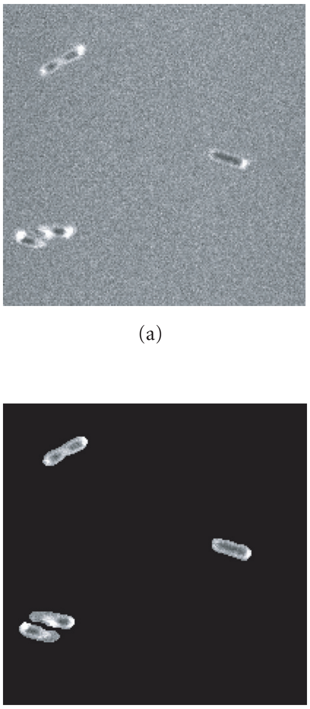

(a)

(b)

Figure 4: Images of *E. coli* cells expressing a GFP-fusion protein (a) before and (b) after fluorescence denoising.

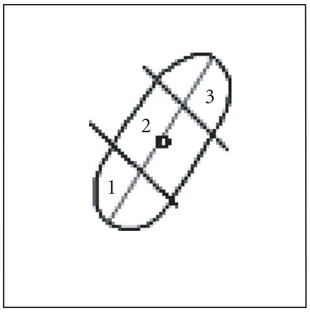

Figure 5: Single cell hypothetically divided into three segments.

that might be present, respectively. A disk-shaped structuring element with radius of 1 pixel is used as a structuring element for these operations.

The clean binary images generated from both DIC and fluorescence image processing are divided into three labeled binary images, containing the cell edge information, the filled contour area of the cells, and the sites of GFP-fusion protein localization. A labeling function provided by MATLAB as *bwlabel* that tags independent groups of objects in the image with a unique label is used.

### 2.3. Feature extraction

A list of relevant features that can be obtained from fluorescence microscopy images of cells is described by Boland and Murphy [11]. We select a subset of features relevant to this study which include the following.

(i) *Number of cells in an image*: calculated by counting the number of labels obtained from the DIC image using the function *bwlabel* provided in MATLAB.

(ii) *Area of individual cells*: calculated by counting the number of pixels under each filled contour label.

(iii) *Area of GFP localization sites within a cell*: calculated by counting the number of pixels of the GFP-localization image within a cell bounded by the cell edge information image and is used for detecting inclusion bodies.

(iv) *Diameter of individual cells*: calculated as the value of the greatest eccentricity, that is, longest distance between any two points in an edge image, or the length of the major axis of the cell.

(v) *Center of gravity (COG) of cells, $COG_c$-($x_c, y_c$), and COG of GFP-fusion protein localizations, $COG_f$-($x_f$, $y_f$)*: calculated by the average location of pixels within the cell or the GFP localization spot.

(vi) *Distance of GFP-fusion protein localization sites from the COG of the cell*: calculated as the Euclidean distance between $COG_c$ and $COG_f$. It provides a quantitative measurement with respect to the distribution of the GFP localization sites within the cell.

(vii) *Number of GFP-fusion protein localization sites within each cell*: extracted by performing an AND operation between the labeled DIC image and the labeled fluorescence image by considering one cell at a time. This gives the number of localization sites within each cell. Ideally, this number would be 2 for growing cells and 3 for dividing cells in our test system. The possibility of other values is discussed in the next section.

### 2.4. Pattern recognition

As mentioned earlier, the decision rule involved in identifying a positive interaction is based on results described in [8, 9]. The presence of two (one at each pole of *E. coli* cells) or three GFP-fusion protein localization sites (both poles and an extra medial localization site) is considered as a positive interaction between the two proteins of interest. The decision rule for an interaction as stated in [8] was used by the expert to interpret a positive interaction. The algorithm applied the same principle by using features that included the position of localization.

The set of features described in Section 2.3 are used to quantify the number of localization sites and their position within the cell. Once the number of GFP-fusion protein localization sites in each cell is identified, the distance between their respective COG's is calculated and compared with the diameter of the cell. We equally segment a cell into three parts along the diameter, where the first and third segments are considered to be the cell poles (Figure 5). Condition for a localization site in the first or third segment is shown in (1) and

Table 1: Examples of GFP-fusion protein localization patterns and features used to derive a positive or negative decision.

| Test case (schematic) | D/6 $\mu$m | Localization sites | d $\mu$m | Decision |
|---|---|---|---|---|
|  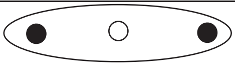 | 0.67 | 1<br>2 | 1.45<br>1.68 | Positive |
|  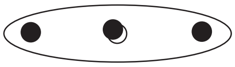 | 0.89 | 1<br>2<br>3 | 2.31<br>0.07<br>2.25 | Positive |
|  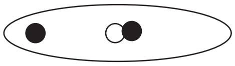 | 0.87 | 1<br>2 | 0.87<br>2.4 | Negative |
|  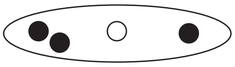 | 2.67 | 1<br>2<br>3 | 3.8<br>5.08<br>6.7 | Negative |
|  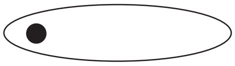 | 0.9 | 1 | — | Negative |

*D: diameter of the cell.*
*d: distance between COG of the cell and localization sites.*

the condition for the same in the second segment is shown in (2):

$$\sqrt{\left[(x_c - x_f)^2 + (y_c - y_f)^2\right]} > \text{Diameter}_c/6, \qquad (1)$$

$$\sqrt{\left[(x_c - x_f)^2 + (y_c - y_f)^2\right]} < \text{Diameter}_c/6, \qquad (2)$$

where $(x_c, y_c)$ and $(x_f, y_f)$ are $\text{COG}_c$ and $\text{COG}_f$, respectively, and $Diameter_c$ is the diameter of the cell. This information regarding location of localization sites within individual cells is used to arrive at a decision regarding a positive interaction. Table 1 shows an example of how the pattern recognition procedure is carried out and how the decision is made. We can see that in order to be identified as a positive interaction, the first criterion is that there must be two or three localization sites within a cell. Based on that, the second criterion follows (1) and (2) for further classification.

### 2.5. Algorithm evaluation

Since the algorithm first identifies the presence of a cell and then identifies the presence of a positive interaction in the identified cell, we have split the evaluation procedure, respectively. Sensitivity of the algorithm to first identify a cell was evaluated followed by the evaluation of sensitivity of the algorithm to identify a positive interaction.

Performance of the algorithm is quantified by comparing its ability to identify positive interactions within a given image to the ground truth (laid down by an expert) of that specific image. With prior knowledge of the decision rule, there was no training data involved. The localization patterns from unprocessed images are studied by an expert in a totally independent event, and cells with positive localization patterns are identified. Decisions on each cell within an image both by the algorithm and the expert are compared, and the number of true positive (TP), false positive (FP), true negative (TN), and false negative (FN) cases is calculated.

Definitions of terms indicated above are as follows:

(i) TP: the cell is identified by the expert and the automated system also identifies the cell;
(ii) TN: no cell is identified by the expert and the automated system does not identify one either;
(iii) FP: no cell is identified by the expert but the automated system identifies one;
(iv) FN: the cell is identified by the expert but the automated system misses it.

Using the terminology in pattern recognition performance evaluation, *sensitivity* is defined as the probability of the system identifying an interaction when one is present, and *specificity* is defined as the probability of the system not identifying an interaction when one is not present. Similarly, for evaluating the algorithm's sensitivity towards identifying a positive interaction within an identified cell, "positive" stands for an identification of a positive protein-protein interaction, while "negative" stands for a negative protein-protein interaction; similar to the cell identification problem, "true" indicates a consistency between the ground truth and the system decision, while "false" represents an inconsistency.

### 3. EXPERIMENTS AND RESULTS

The test images used for evaluating the automated image analysis consist of a set of 16 DIC and corresponding fluorescence images. These were captured over 2 experiments carried out on different samples and imaged at different magnifications and cell population (a total of about 390 cells) in order to avoid any amount of bias in the procedure. Two problems are addressed in the automated system. Identifying individual cell contours to quantify cell count is the

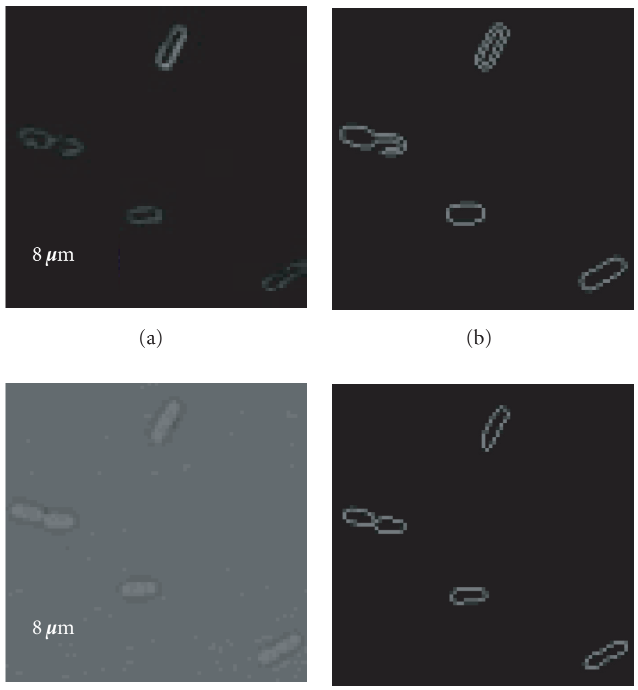

(a) (b) (c) (d)

Figure 6: Comparison of edge detection from images of *E. coli* cells stained with a membrane dye and from DIC images. (a) *E. coli* cell membranes stained with FM5-95. (b) Results of edge detection using the image in (a). (c) DIC image of *E. coli* cells. (d) Results of edge detection using the image in (c).

first problem, and is obtained by using the DIC image. The second problem is to use the fluorescence image to determine if the identified cells have positive localization patterns.

### 3.1. The image processing algorithm evaluation

The essential information obtained from the DIC image is the cell boundary. Experimentally, the cell boundaries can be visualized by several techniques, such as the use of a membrane dye and the DIC image. For this analysis, the use of a DIC image is chosen over the use of images of membrane-staining dyes.

Although the use of images with stained membranes gave a fair indication of the cell boundaries for isolated cells (Figures 6(a), 6(b)), a similar analysis for a clump of cells produced inconsistent results (data not shown). The cell boundary becomes difficult to extract when there are overlapping cells or cells that lie in close proximity to each other. Care is taken during image acquisition to avoid fields of overlapping cells. In addition, objects in the image with very large (greater than twice the mean area) or very small (less than one-third of the mean area) areas can lead to ambiguous results and thus are eliminated to provide more meaningful results. Because DIC images typically result in a thick cell boundary, cells in close proximity were distinguished from each other using the inner boundary contour. During the process of DIC image analysis, a difference image is first generated from the original image and its bottom-hat version to provide an improvement in the contrast along boundaries in the case of closely spaced cells. A high-pass second-order Butterworth filter in the frequency domain is then applied on this difference image to enhance the high-frequency (mostly edge-based) information that improves segmentation results. This step is followed by adaptive histogram equalization. The resulting image is then processed through an averaging filter to avoid oversegmentation.

The *Canny* filter is chosen to segment the edges from the processed DIC image over other algorithms that include the *Sobel* filter and the active contour algorithm [15].

The DIC image shows a thick boundary to the cells, thus producing a ring-like binary image. Upon observation that the inner side of the ring leads to more consistent boundary determination, the weak outer edges are discarded by keeping about 15% of the lowest intensity value using a high threshold in the *Canny* detector (Figures 6(c), 6(d)).

While performing morphological operations, particular attention is taken in choosing an appropriate structuring element. The shape and size of the structuring element are defined by the object shape under study. Since these cells possess smooth corners, a *disk*-shaped structuring element is employed and a radius of 1 pixel is chosen, taking into consideration the spatial dimensions (in pixels) of the cell.

### 3.2. The inclusion bodies identification

Once the cell boundaries are extracted, the second problem is to use the fluorescence image to identify and label the sites of GFP-fusion protein localization. Identification of a positive protein-protein interaction using the DivIVA assay is based on recruitment of the GFP-fusion protein to the cell poles following expression of the DivIVA-fusion protein [8, 9]. Experimentally, DIC and fluorescence images are collected from cells expressing the GFP-fusion protein before and after induction of the DivIVA-fusion protein. The expected result is that the GFP-fusion protein will localize diffusely throughout the cell *before* induction and to the cell poles *after* induction of the DivIVA-fusion protein if there is an interaction between the two proteins being tested. However, these expected results can be complicated by the presence of “inclusion bodies” caused by overexpression of the GFP-fusion protein in bacterial cells. The aggregates of overexpressed GFP-fusion protein tend to localize to the cell poles thereby mimicking the localization pattern produced by a positive protein-protein interaction. This is an experimental problem inherent to the biological system under study and complicates the automation of image analyses.

To distinguish between inclusion bodies and positive interactions, we have employed the experimental solution of identifying inclusion bodies in the sample before expression of the DivIVA-fusion protein. The image processing block detailed in Section 2.2 is applied to both the DIC and fluorescence images acquired before induction and three labeled images are generated. Based on the labeled images, the percentage area occupied by the localized fluorescence within each cell is calculated, which after experimentation

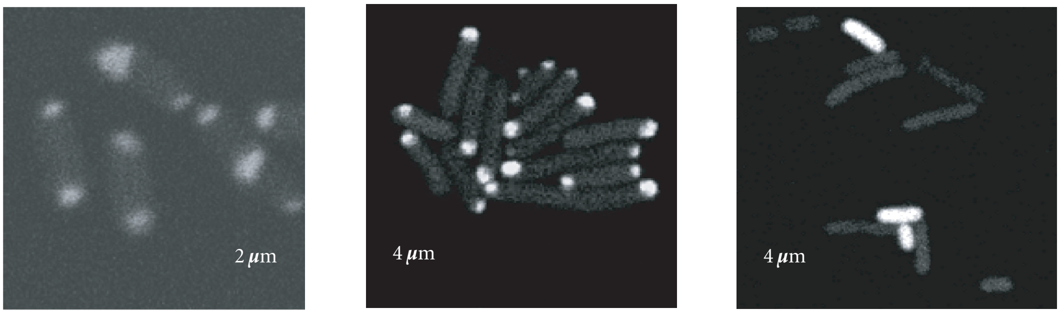

(a) (b) (c)

Figure 7: Examples of localization patterns displayed by *E. coli* cells expressing GFP-fusion proteins. (a) Cells with GFP-fusion protein localization at the poles corresponding to a positive protein-protein interaction. (b) Cells displaying inclusion bodies before induction of the DivIVA-fusion protein. (c) Cells showing cytoplamic GFP-fusion protein localization before induction. Note the visual similarity between the image in (a) and (b).

and observation is found to be less than 60% for images with inclusion bodies. Thus, if inclusion bodies are present before induction of the DivIVA-fusion protein, the sample will not be further analyzed. Representative images of bacterial cells displaying a diffuse GFP-fusion protein localization pattern, inclusion bodies, or a positive protein-protein interaction are shown in Figure 7.

### 3.3. The pattern recognition evaluation

Following the elimination of samples displaying inclusion bodies and the induction of the DivIVA-fusion protein, a second set of DIC and fluorescence images is acquired. The number of distinct protein localization sites inside a given cell is then quantified to determine whether the results are consistent with a positive protein-protein interaction. Both nondividing and dividing cells can reveal patterns of positive interaction. In nondividing cells, a positive result is characterized by localization of the GFP-fusion protein at both cell poles (2 sites). In dividing cells, a positive result is determined by localization of the GFP-fusion protein to the cell poles and also to the center of the cell since DivIVA is known to localize to the medial region during cell division (3 sites) [9].

The statistical features extracted from data using various algorithms discussed in Section 2.3 are used to characterize a set of 16 sample test images. Results from one set of images are shown in Figure 8.

The final image (Figure 8(e)) shows individual features in different color channels generated by pseudocoloring the target locations. In this particular image set, 13 cells are present.

All of the nondividing cells (7 in total) displayed a pattern of GFP-fusion protein localization consistent with a positive interaction (localization at both poles). The remaining cells in the image are undergoing division and all but one display sites of GFP-fusion protein localization at both poles and the medial region of the cell. In this test case, the algorithm identified 12 positive cells out of 13 total cells, consistent with results obtained by an expert scorer.

The number of GFP-fusion protein localization sites and their respective positions within individual cells is used to identify cells with positive interaction patterns. The performance of the automated system in identifying individual cells and in identifying positive interacting cells is evaluated, respectively. For the first case, only a single FP was observed, thereby producing a specificity of 1 for 15 images and a mean specificity of about 0.9995 ($\sim 1$) over the entire dataset. The single FP was due to an image field containing cell debris that was not eliminated by the mean area-based filter and was therefore counted as a cell.

The free parameter used in the procedure of identifying a cell is the $\sigma$, used in the Canny filter (Section 2.2). In Figure 9, we use sensitivity to illustrate the performance of the automated system, and evaluate the effect of the free parameter $\sigma$, used in the Canny filter (Section 2.2). We observe that except for one image, a choice of $\sigma = 0.85$ generates the highest sensitivity, averaged at 86% with the smallest standard deviation of 0.11, indicating the best robustness.

Similar to the previous evaluation procedure, sensitivity and specificity of the algorithm to identify a positive interaction within an identified cell was calculated for each image. Mean sensitivity and specificity values were then calculated over the entire dataset. From the 16-image dataset, again only one image contained a false positive case, thereby producing a specificity of 1 for all but one case (image). The mean specificity of the algorithm over the entire dataset was thus found to be about 0.9989 ($\sim 1$). Such false positives can be attributed to the presence of inclusion bodies in the cells, which localized in a pattern similar to that of a true positive interaction. Although our experimental design reduces the number of images collected that show inclusion bodies, this possibility cannot be completely eliminated. During the process of recognizing interactions, the threshold applied on the fluorescence image was another free parameter used and its effect on the sensitivity of positive interaction identification within a cell is evaluated, as shown in Table 2. A 4-fold cross-validation was performed on the 16 images in order to eliminate biased results. A threshold value equal to one-thirds of that obtained by using the *Otsu* method was observed to produce the highest sensitivity with the smallest average standard derivation of 0.0959, indicating the best robustness. Thus, the average sensitivity of the algorithm to

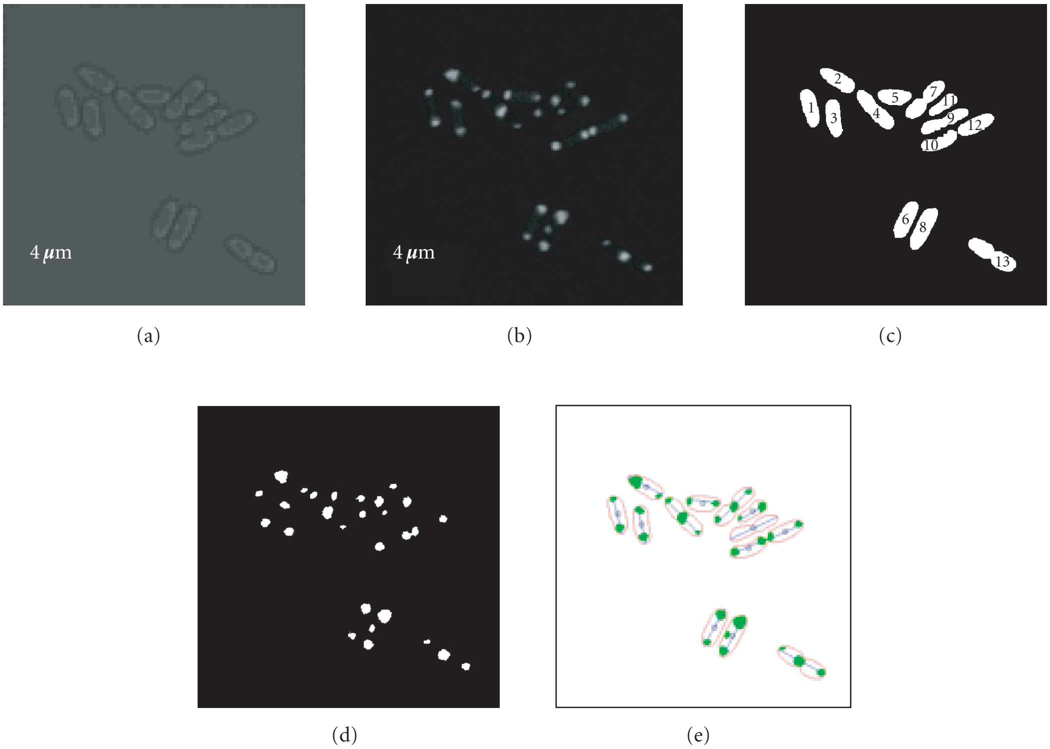

FIGURE 8: Image processing steps leading to a final pseudocolored image from DIC and fluorescence images of *E. coli* cells expressing a GFP-fusion protein. (a) Original DIC image. (b) Original fluorescence image. (c) Binary DIC image. (d) Binary GFP image before labeling. (e) Pseudocolored image showing cell boundaries (red), cell diameter (blue), sites of GFP-fusion protein localization (green), and the COG of individual cells (black).

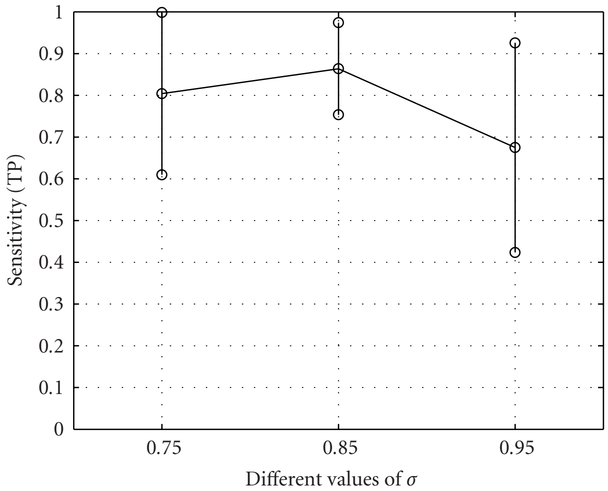

FIGURE 9: Effect of the different $\sigma$ values in the *Canny* edge detector on the sensitivity of system towards identifying a cell. Mean sensitivity derived from 16 testing images: 0.8 when $\sigma = 0.75$; 0.86 when $\sigma = 0.85$; and 0.67 when $\sigma = 0.95$.

TABLE 2: Four-fold cross-validation results to determine the optimum threshold value (STD—standard deviation). Mean sensitivities towards identifying a positive interaction derived from 16 testing images: 0.57 when using the threshold value ($x$) from Otsu's method; 0.7 when using $x/2$ as the threshold; 0.84 when using $x/3$ as the threshold, and 0.58 for a threshold of $x/4$.

| | | 1 | 2 | 3 | 4 |
|---|---|---|---|---|---|
| $x$ | Sensitivity | 0.4924* | 0.5654 | 0.5078 | 0.7181 |
| | STD | 0.0992 | 0.0869 | 0.2092 | 0.1362 |
| $x/2$ | Sensitivity | 0.6803 | 0.6939 | 0.7078 | 0.7181 |
| | STD | 0.1119 | 0.1206 | 0.1347 | 0.1831 |
| $x/3$ | Sensitivity | 0.8388 | 0.8140 | 0.8563 | 0.8667 |
| | STD | 0.0744 | 0.1085 | 0.0760 | 0.1247 |
| $x/4$ | Sensitivity | 0.4894 | 0.4105 | 0.7174 | 0.7267 |
| | STD | 0.2801 | 0.3967 | 0.1662 | 0.1640 |

*: mean sensitivity of the algorithm to identify a positive interaction at a threshold of "$x$" and the first four images as a testing dataset.

identify a cell in an image was found to be 0.863 and the average sensitivity of the algorithm to determine the presence of positive localization patterns in the identified cell was found to be about 0.8439.

### 4. DISCUSSION AND CONCLUSION

Identifying protein-protein interactions is critical for understanding the function of proteins in cells and provides a framework for understanding biological networks.

As the field of proteomics expands, comprehensive studies of protein-protein interactions within an organism are becoming possible. One obstacle in these studies is the difficulty in processing large datasets, especially those containing large sets of fluorescence images.

In this study, we described an algorithm that can be exploited for high-throughput screening of protein-protein interactions in bacterial cells based on localization patterns. We developed an automated image analysis package that can quantify the number of cells in an image, recognize protein localization patterns in individual cells, and produce a statistical output to quantify the number of cells displaying a specific localization pattern. Unique solutions to solve problems due to the ambiguity arising from adjoining cells, inclusion bodies, and the problems caused by background fluorescence were offered.

Different edge detection techniques were tried and tested to identify cell boundaries. The Canny edge detector [16] was used to obtain cell contours in the segmentation process as it was a simpler, faster, and more effective method in this case, compared to active contours [15], which is a popular technique in medical image analysis. Care was taken to remove unwanted information (weak edges) and retain strong edge information by varying $\sigma$. A very small value of $\sigma$ resulted in the inclusion of weak edges and a very high value resulted in the loss of actual edge information.

A simple thresholding technique was used to segment localization sites in fluorescence images. Results for different threshold values were compared with one another. The choices of parameters for all morphological operations were made in accordance with the resolution of images. Care was taken to avoid overlapping of closely spaced cells in the final image.

In the DivIVA-based interaction assay, overexpression of the GFP-fusion protein can lead to the formation of inclusion bodies, which have a tendency to accumulate at the poles of *E. coli* cells and look very similar to the sites of GFP-fusion protein localization associated with a positive protein-protein interaction. In order to reduce false positive cases, experimental testing for inclusion bodies was conducted before computationally based assessment of subcellular protein localization. This problem is specific to this particular assay and may not be a consideration for other types of cells, labels, or protein localization experiments. However, this potential obstacle illustrates the importance of integrating image acquisition and analysis with experimental design.

Identified cells are considered true positives, and cells missed by the algorithm are considered as false negatives. These definitions are used to calculate the sensitivity of the algorithm to identify individual cells. Similarly, cells properly identified by the algorithm are labeled as positive or negative results by an expert in accordance with the decision rule discussed above. When these results are compared with the results obtained by the algorithm, we arrive at true positive and false negative values that help us calculate the sensitivity of the algorithm to identify positive localization patterns.

For this study, we used a DivIVA-based assay to test two well characterized proteins that are known to interact. Low sensitivities in a few cases can be attributed to a number of experimental and biological factors such as the focal plane of the collected image and plasmid loss. In this situation, the performance of the algorithm is acceptable since the final output is a binary decision (there is or is not an interaction between the proteins of interest). In practice, a threshold level of 50% or more positive cells is considered a positive interaction based on studies of pairs of known interacting proteins [10].

Although the automated system was tested and evaluated on sample images from a DivIVA-based interaction screen in which cells display very specific localization patterns [8, 9], it could be adaptable to a wider range of experimental studies, involving multiple fluorescent labels or other imaging modalities with slight modifications. Such a system can also be employed to reduce the size of image datasets by selecting those that possess desired features, such as positive interactions or specific localization patterns.

In summary, from the set of 16 images, the automated system achieves, on average, 86% sensitivity in cell identification and 84% sensitivity in identifying positive localization patterns in cells. In addition, according to studies in [10], an identification of at least 50% positive cells in an image is sufficient to indicate a positive interaction between the two proteins assessed in the assay. Based on this criterion, the automated system presents 100% accuracy in the identification of positive interactions in this dataset.

### ACKNOWLEDGMENTS

This research was funded by the US DOE Office of Biological and Environmental Sciences Genomics: GTL program. Oak Ridge National Laboratory is managed by UT-Battelle, LLC, for the US Department of Energy under Contract no. DE-AC05-00OR22725.

### REFERENCES

[1] M. V. Boland and R. F. Murphy, "After sequencing: quantitative analysis of protein localization," *IEEE Engineering in Medicine and Biology Magazine*, vol. 18, no. 5, pp. 115–119, 1999.

[2] D. W. Shattuck and R. M. Leahy, "Automated graph-based analysis and correction of cortical volume topology," *IEEE Transactions on Medical Imaging*, vol. 20, no. 11, pp. 1167–1177, 2001.

[3] B. W. Reutter, G. J. Klein, and R. H. Huesman, "Automated 3-D segmentation of respiratory-gated PET transmission images," *IEEE Transactions on Nuclear Science*, vol. 44, no. 6, part 2, pp. 2473–2476, 1997.

[4] T. N. Davis, "Protein localization in proteomics," *Current Opinion in Chemical Biology*, vol. 8, no. 1, pp. 49–53, 2004.

[5] C. A. Glasbey, "Problems in digital microscopy," in *Proceedings of 18th International Biometric Conference (IBC '99)*, pp. 183–200, Amsterdam, The Netherlands, 1999.

[6] J. H. Price, A. Goodacre, K. Hahn, et al., "Advances in molecular labeling, high throughput imaging and machine intelligence portend powerful functional cellular biochemistry tools," *Journal of Cellular Biochemistry*, vol. 87, no. 39 supplement, pp. 194–210, 2002.

[7] D. H. Edwards and J. Errington, "The Bacillus subtilis DivIVA protein targets to the division septum and controls the site specificity of cell division," *Molecular Microbiology*, vol. 24, no. 5, pp. 905–915, 1997.
[8] Z. Ding, Z. Zhao, S. J. Jakubowski, A. Krishnamohan, W. Margolin, and P. J. Christie, "A novel cytology-based, two-hybrid screen for bacteria applied to protein-protein interaction studies of a type IV secretion system," *Journal of Bacteriology*, vol. 184, no. 20, pp. 5572–5582, 2002.
[9] B. D. Corbin, B. Geissler, M. Sadasivam, and W. Margolin, "Z-ring-independent interaction between a subdomain of FtsA and late septation proteins as revealed by a polar recruitment assay," *Journal of Bacteriology*, vol. 186, no. 22, pp. 7736–7744, 2004.
[10] J. L. Morrell-Falvey and M. J. Doktycz, "High throughput imaging-based assay for detecting protein interactions in microbial cells," manuscript in preparation.
[11] M. V. Boland and R. F. Murphy, "A neural network classifier capable of recognizing the patterns of all major subcellular structures in fluorescence microscope images of HeLa cells," *Bioinformatics*, vol. 17, no. 12, pp. 1213–1223, 2002.
[12] L.-M. Guzman, D. Belin, M. J. Carson, and J. Beckwith, "Tight regulation, modulation, and high-level expression by vectors containing the arabinose P(BAD) promoter," *Journal of Bacteriology*, vol. 177, no. 14, pp. 4121–4130, 1995.
[13] F. W. Larimer, P. Chain, L. Hauser, et al., "Complete genome sequence of the metabolically versatile photosynthetic bacterium *Rhodopseudomonas palustris*," *Nature Biotechnology*, vol. 22, no. 1, pp. 55–61, 2004.
[14] N. Otsu, "Threshold selection method from gray-level histograms," *IEEE Transactions on Systems, Man, and Cybernetics*, vol. 9, no. 1, pp. 62–66, 1979.
[15] C. Xu and J. L. Prince, "Snakes, shapes, and gradient vector flow," *IEEE Transactions on Image Processing*, vol. 7, no. 3, pp. 359–369, 1998.
[16] J. Canny, "Computational approach to edge detection," *IEEE Transactions on Pattern Analysis and Machine Intelligence*, vol. 8, no. 6, pp. 679–698, 1986.

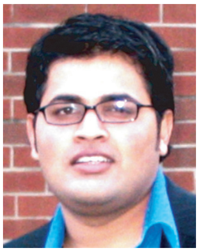

**S. Venkatraman** is currently a Research Associate at the Oak Ridge National Laboratory as a part of the Biological and Nanoscale Systems Group. He received his Master's of Sciences degree in electrical engineering from The University of Tennessee, Knoxville in 2005. He did his Bachelor's of Engineering in the field of electronics and instrumentation from Muffakham Jha College of Engineering Technology. Most of his research is focused towards image processing/analysis for biomedical applications. His current research interests involve automated AFM image analysis and data fusion. Apart from image processing, pattern recognition, and computer vision, he also has interests in biomedical and nanoscale instrumentation.

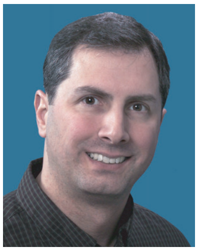

**M. J. Doktycz** is a Senior Staff Scientist and Program Leader for Biomedical and Biophysics Programs in the Life Sciences Division at the Oak Ridge National Laboratory. He received his B.S. degree in biology and chemistry in 1985 and Ph.D. degree in chemistry in 1991 from the University of Illinois at Chicago. His research interests focus on the intersection of biological and nanoscale systems. His laboratory is involved in the development of analytical technologies for postgenomics studies with specific emphases on molecular and cellular imaging techniques and the use of nanomaterials to study and mimic biological systems.

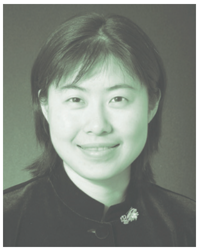

**H. Qi** received her Ph.D. degree in computer engineering from North Carolina State University in 1999, B.S. and M.S. degrees in computer science from Northern JiaoTong University, Beijing, China in 1992 and 1995, respectively. She is now an Associate Professor in the Department of Electrical and Computer Engineering at the University of Tennessee, Knoxville. Her current research interests are advanced imaging and collaborative processing in sensor networks, hyperspectral image analysis, and bioinformatics. She has published over 70 technical papers in archival journals and refereed conference proceedings, including a co-authored book in machine vision. She is the recipient of the NSF CAREER Award and Chancellor's Award for Professional Promise in Research and Creative Achievement. She serves on the editorial board of Sensor Letters and is the Associate Editor for Computers in Biology and Medicine. She coedited a special issue on distributed sensor networks for real-time systems with adaptive reconfiguration of Journal of Franklin Institute. She is a Senior Member of IEEE and an Associate Member of Sigma Xi.

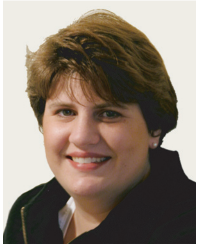

**J. L. Morrell-Falvey** received her Ph.D. degree in genetics from Purdue University in 1997 and her B.A. degree from Saint Mary's University in Minnesota in 1991. She is now a Staff Scientist in the Life Sciences Division at Oak Ridge National Laboratory. Her current research focuses on the use of genomic and live cell imaging tools to study microbial systems.

